# Expression of concern: Optimized Cu-doping in ZnO electro-spun nanofibers for enhanced photovoltaic performance in perovskite solar cells and photocatalytic dye degradation

**DOI:** 10.1039/d4ra90153c

**Published:** 2024-12-23

**Authors:** Kang Hoon Lee, Rabeea Farheen, Zafar Arshad, Mumtaz Ali, Hamza Hassan, Mubark Alshareef, A. Dahshan, Usama Khalid

**Affiliations:** a Department of Energy and Environment Engineering, The Catholic University of Korea 43-Jibong-ro Bucheon-si 14662 Republic of Korea; b Department of Physics, Government College Women University Faisalabad Pakistan; c School of Engineering and Technology, National Textile University Faisalabad Pakistan zafarnubii@gmail.com; d Department of Chemical Engineering, University of Engineering and Technology Peshawar Pakistan; e Department of Chemistry, Faculty of Applied Science, Umm Al Qura University Makkah 24230 Saudi Arabia mmshreef@uqu.edu.sa; f Department of Physics, College of Science, King Khalid University Abha Saudi Arabia; g Department of Organic and Nano Engineering, Hanyang University 222 Wangsimni-ro, Seongdong-gu Seoul 04763 Republic of Korea

## Abstract

Expression of concern for ‘Optimized Cu-doping in ZnO electro-spun nanofibers for enhanced photovoltaic performance in perovskite solar cells and photocatalytic dye degradation’ by Kang Hoon Lee *et al.*, *RSC Adv.*, 2024, **14**, 15391–15407, https://doi.org/10.1039/D4RA01544D.

*RSC Advances* is publishing this expression of concern in order to alert readers that concerns have been raised regarding the integrity of the XRD patterns in Fig. 2a, the Raman spectra in Fig. 2b, the SEM data in Fig. 3, the FTIR spectra in Fig. 4a and b, and the Tuac plot in Fig. 5b. An expression of concern will continue to be associated with the article until a conclusive outcome is reached.

Laura Fisher

9th December 2024

Executive Editor, *RSC Advances*

